# Personalise antidepressant treatment for unipolar depression combining individual choices, risks and big data (PETRUSHKA): rationale and protocol

**DOI:** 10.1136/ebmental-2019-300118

**Published:** 2019-10-23

**Authors:** Anneka Tomlinson, Toshi A Furukawa, Orestis Efthimiou, Georgia Salanti, Franco De Crescenzo, Ilina Singh, Andrea Cipriani

**Affiliations:** 1 Department of Psychiatry, University of Oxford, Oxford, UK; 2 Graduate School of Medicine Faculty of Medicine, Kyoto University, Kyoto, Japan; 3 Institute of Social and Preventive Medicine, University of Bern, Bern, Switzerland; 4 Oxford Health NHS Foundation Trust, Warneford Hospital, Oxford, UK

**Keywords:** depression and mood disorders, adult psychiatry

## Abstract

**Introduction:**

Matching treatment to specific patients is too often a matter of trial and error, while treatment efficacy should be optimised by limiting risks and costs and by incorporating patients’ preferences. Factors influencing an individual’s drug response in major depressive disorder may include a number of clinical variables (such as previous treatments, severity of illness, concomitant anxiety etc) as well demographics (for instance, age, weight, social support and family history). Our project, funded by the National Institute of Health Research, is aimed at developing and subsequently testing a precision medicine approach to the pharmacological treatment of major depressive disorder in adults, which can be used in everyday clinical settings.

**Methods and analysis:**

We will jointly synthesise data from patients with major depressive disorder, obtained from diverse datasets, including randomised trials as well as observational, real-world studies. We will summarise the highest quality and most up-to-date scientific evidence about comparative effectiveness and tolerability (adverse effects) of antidepressants for major depressive disorder, develop and externally validate prediction models to produce stratified treatment recommendations. Results from this analysis will subsequently inform a web-based platform and build a decision support tool combining the stratified recommendations with clinicians and patients’ preferences, to adapt the tool, increase its’ reliability and tailor treatment indications to the individual-patient level. We will then test whether use of the tool relative to treatment as usual in real-world clinical settings leads to enhanced treatment adherence and response, is acceptable to clinicians and patients, and is economically viable in the UK National Health Service.

**Discussion:**

This is a clinically oriented study, coordinated by an international team of experts, with important implications for patients treated in real-world setting. This project will form a test-case that, if effective, will be extended to non-pharmacological treatments (either face-to-face or internet-delivered), to other populations and disorders in psychiatry (for instance, children and adolescents, or schizophrenia and treatment-resistant depression) and to other fields of medicine.

## Introduction

Mental disorders are a major cause of the global health burden, accounting for 23% of years lived with disability.^1^ With 350 million people affected in the world, depressive disorder is considered to be the second leading cause of the global health burden.^2^ The burden for major depressive disorders is largely due to significant deficits in treatment provision.^3^ There are a number of pharmacological and non-pharmacological efficacious interventions available for major depressive disorder and the key challenge is how best to implement these treatments in clinical practice.^4^ About 80% of people identified as suffering from depressive disorder in primary care in the UK receive an antidepressant prescription in the first year of diagnosis.^5^ However, the majority of prescriptions are for less than 30 days,^5^ while an adequate trial of antidepressants is generally recommended to be 6–8 weeks before changing or stopping the medication. Too short a duration of treatment both limits the therapeutic effect^6^ and increases the risk of withdrawal symptoms because antidepressants should be tapered off over a period of 4 weeks or more.

While a number of factors contribute to suboptimal treatment durations, two of the most prominent ones are the initial side effects of the medication and their perceived marginal efficacy. These factors are exacerbated by our current inability to predict which drug(s) will cause side effects in which patients, and which drug(s) will work most effectively for which patients. Better means of tailoring treatment to individuals are urgently needed. This has been recognised by important institutions (for instance, the National Institute for Health and Care Excellence in the UK or the Royal Australian and New Zealand College of Psychiatrists in Australia), although there are no reliable ways of doing so, to the best of our knowledge.^7^ Over the last few years, however, there has been a substantial accumulation of clinical trial data as well as long-term outcomes from ‘real-world’ datasets. The wealth of this available data, together with the development of new methods in evidence synthesis, has created major opportunities to improve patient outcomes by using existing therapies more efficiently, in a more targeted manner.^8^ Recent models have been able to predict the probability of response for a specific subgroup of patients with depressive disorder or estimate the chances that an individual will have a particular side effect.^9^ By identifying and then matching individual antidepressants to individual patients, clinicians can more precisely customise treatment to patients’ needs and thus improve their outcome.^10^

Despite recent progress in this field,^11^ psychiatry continues to lag behind other specialties like cardiology, oncology, immunology and neurology. Currently, no validated system of tailoring treatment choices is available. Indeed, matching treatment to specific patients is too often a matter of trial and error, while treatment efficacy should be optimised by limiting risks and costs and by incorporating patients’ preferences.^10^ A joint analysis of individual participant data obtained from multiple clinical trials as well as real-world studies, has the potential to provide ‘personalised’ estimates of comparative effectiveness, but also to provide ‘individualised’ predictions regarding the probability of response to treatment and of experiencing side effects.^4^ Factors influencing an individual’s drug response in depressive disorder may include a number of clinical variables (such as number and types of previous treatments, severity of illness, concomitant anxiety etc) as well demographics (for instance, age, weight, social support and family history).^4^

However, the wealth and variety of all possible such factors create its own challenges and new approaches are needed. These approaches should^12^:

- Be based on robust evidence.
- Be acceptable to patients and clinicians.
- Guide treatment personalisation, incorporating patients’ views and preferences and clinical judgement.^13^
- Support probabilistic decision-making.^14^

**Figure 1:**
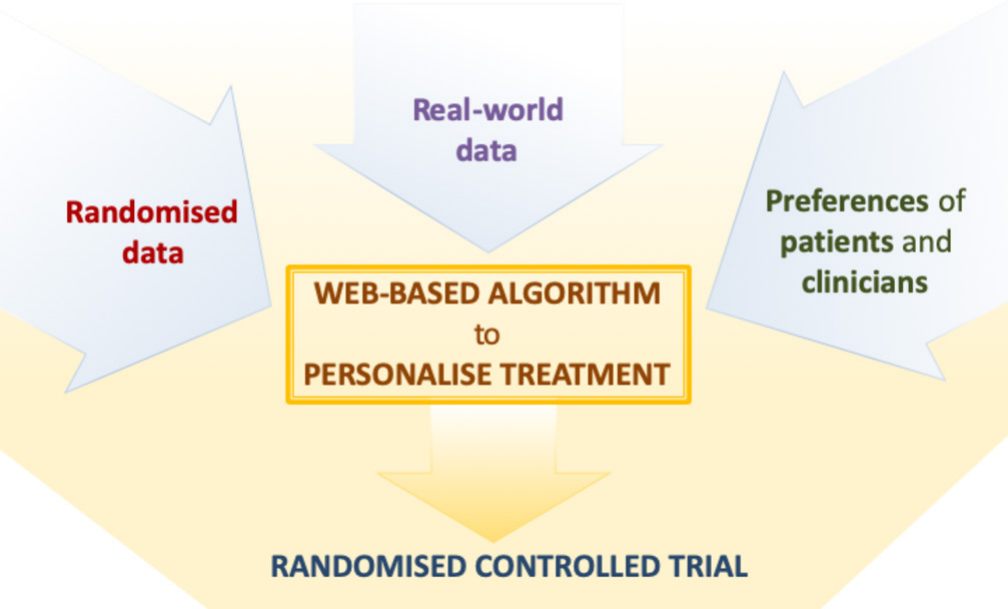
Overview of the project to develop and validate the personalise antidepressant treatment for unipolar depression combining individual choices, risks and big data treatment algorithm. Evidence from individual patient data from antidepressant trials in major depressive disorder, in combination with real-word (observational) data and patient and clinician preferences (phase I), will be used to develop a web-based personalised treatment algorithm (phase II). The web-based algorithm will be tested in a multicentre, pragmatic, randomised controlled trial comparing the treatment algorithm with usual care (Phase 3).

Our project, funded by the National Institute of Health Research, is aimed at developing and subsequently testing a precision medicine approach to the pharmacological treatment of major depressive disorder, which can be used in everyday clinical settings. To this aim, we will jointly synthesise data from patients with depressive disorder, obtained from diverse datasets, including both randomised trials as well as observational, real-world studies. Results from this analysis will subsequently inform a web-based platform, which will allow shared decision-making at the individual-patient level during the routine consultation between clinicians and patients (see figure 1).

## Methods and analysis

This project is called ‘personalise antidepressant treatment for unipolar depression combining individual choices, risks and big data’ (PETRUSHKA) and its overarching objectives are to:

1. Summarise the highest quality and most up-to-date scientific evidence about comparative effectiveness and tolerability (adverse effects) of antidepressants for major depressive disorders in adults, develop and externally validate prediction models to produce stratified treatment recommendations.^15^
2. Build a decision support tool combining the stratified recommendations with clinicians and patients’ preferences, to adapt the tool, increase its reliability and tailor treatment indications to the individual-patient level.
3. Test whether use of the tool relative to treatment as usual in real-world clinical settings leads to enhanced treatment adherence and response, is acceptable to clinicians and patients, and is economically viable.

To achieve these objectives, the project is structured in three phases: (1) development and validation of the models that predict clinical outcomes for patient subgroups and different treatments, (2) building a treatment algorithm, (3) designing and conducting a pragmatic randomised trial to evaluate the usefulness of the algorithm. Patients and clinicians will be involved throughout the project to understand preferences and values in relation to clinical outcomes; and acceptability of and trust in the decision support tool.

### ​Phase I: development of prediction models, using randomised and real-world evidence

When several treatment options are available, standard meta-analyses provide only partial information on relative treatment effects because they can only compare two treatments at a time.^16^ This fragmented approach does not support optimal clinical decision making^17^ and the need for a robust method to summarise evidence across several interventions has been increasingly recognised.^18^ Network meta-analysis (NMA) has been developed to allow the estimation of the relative effect of many treatments one against the other and produce ranked treatment options.^19^

In this project, we will use aggregate and individual-patient data (IPD) from multiple sources, combining methods from IPD network meta-regression and prognostic modelling.^20^ Our aim will be to predict the effects (in terms of efficacy, acceptability and tolerability) of different antidepressants given information on patient-level characteristics. Our models will incorporate a range of possible prognostic factors and effect modifiers. We will use information on both demographic (ie, gender, age, ethnicity, marital status, childhood trauma) as well as clinical characteristics (ie, family history, previous episodes, past treatments, age at onset, duration of index episode, severity, treatment dose, concomitant medication, comorbidity).^21^ We will explore a range of alternative modelling approaches, incorporating evidence from both randomised and observational evidence, utilising and further extending recently proposed methods.^22^ In addition, we will explore advanced machine learning techniques for prognostic modelling, such as artificial neural networks and support vector machines.^23^ We will finally choose the best performing models for each outcome using extensive cross-validation.

The randomised studies selected for the IPD analysis will follow the same selection criteria as our previously published NMA based on aggregate data.^24^ The review protocol with all the methodological details has already been published.^25^ We are aware that not all of the studies selected for inclusion will have data available at the individual patient level; however, we will systematically request access by contacting study authors and/or sponsors and by submitting applications via online platforms. To complement the randomised data and to also collect information about long-term outcomes (especially adverse events), we will use large observational datasets. Real-world routinely collected data may be more representative of the patient population seen in everyday clinical practice. These data will contribute by providing relevant information on the natural course of the condition and the prevalence of various important patient characteristics. We aim to use data from both primary care (QResearch - https://www.qresearch.org/), as well secondary care (UK Clinical Record Interactive Search).^26^

After model development, in order to externally validate all of our models we will use a part of the observational data as ‘ground truth’ test data (‘held-out data’). We will select a specific subgroup of the data, for example, data collected in a specific geographic location and use this approach to test our models, that is, to assess their predictive performance in real-world settings. Overall, our approach aims to harness the strengths of both randomised and observational data.

### ​Phase 2: development of the treatment algorithm

We will then develop a clinical support system (*treatment algorithm*), to guide clinicians, patients and carers in the shared process of decision-making in routine care. Treatment algorithms have contributed to advances in many fields of medicine and psychiatry,^27^ and computerised decision systems have been developed to provide ongoing assistance to clinicians.^28^ However, existing algorithms in psychiatry lack the ability to apply the best knowledge directly to the individual patient and selectively provide information relevant to the characteristics and circumstances of that patient in their specific situation.

We will develop a web-based algorithm that will:

- Utilise the predictions from the predictive models developed in phase I.
- Incorporate preferences and values of clinicians and patients to identify the desired clinical outcome,^29 30^ considering both efficacy and adverse events (for instance, some patients may want to avoid sedation while others tremor or sexual dysfunction).
- Generate the corresponding ranking list of personalised treatment recommendations that will inform the clinical discussion between clinicians and patients, and the final treatment decision.

The algorithm will be implemented in the form of a web application, accessible from any computer or tablet. The input of the algorithm will be a patient’s individual characteristics and preferences. The output will be the models’ estimates regarding the expected outcomes under each treatment, and a proposed treatment ranking according to the patients’ preferences.

During the development of the algorithm, we will organise a series of focus groups with patients, carers and clinicians to gather feedback about the layout of the decision tool, its clinical value, practical utility, and ways to improve it.

### Phase 3: randomised trial

We will conduct a two-arm, multicentre, open-label, randomised controlled trial comparing the treatment algorithm with care as usual. The primary objective of the trial is to determine whether using the treatment algorithm to identify a ‘personalised’ antidepressant results in an increased proportion of patients who keep taking the allocated treatment at 8 weeks, in comparison to care as usual. The secondary objectives are to: (1) assess whether using the treatment algorithm results in greater reduction of depressive symptoms and better adherence to treatment in the long-term; (2) determine the impact on societal costs and cost-effectiveness/cost-utility of the treatment algorithm; (3) explore how the treatment algorithm is used by various stakeholders (patients, carers, clinicians), what they think about it and the impact this has on care and care processes, in order to refine its future implementation in the UK and across different countries.

The treatment algorithm described in phase II will be the intervention, and the control condition will be treatment as usual (ie, clinician prescribing antidepressants without the use of the algorithm). In terms of the study population, we will include males or females, aged 18 years old or above, with a diagnosis of a major depressive episode (either first episode or recurrent) according to standardised criteria and requiring treatment with antidepressant as monotherapy, who are able to provide informed consent. Patients with treatment resistant depression, bipolar disorder, psychosis, substance abuse or current suicidal ideation and those requiring urgent mental healthcare or admission will be excluded.

Participants will be recruited from clinical services (general practitioner surgeries or outpatient clinics) in centres across the UK. Patients with major depressive disorder will be considered for the study if the clinician decides to prescribe an antidepressant to treat the depressive disorder. Participants will be provided with a brief explanation of the study and a baseline visit will be scheduled. The majority of the recruitment is expected to be in primary care as this is where patients with depressive disorder are seen most often; however, patients from secondary care will also be included in order to increase the generalisability of the findings from this trial.

The primary outcome will be acceptability of antidepressant treatment, measured as the number of participants stopping treatment (or changing medication) by week 8 (*all-cause discontinuation*). This measure integrates patients’ and clinicians’ judgments of efficacy and tolerability into a global measure of effectiveness.^24^

Secondary outcomes will focus on both short-term and long-term outcomes, including self-rated change in depressive symptoms measured via remote monitoring, observer-rated change in depressive symptoms, discontinuation due to adverse events only, tolerability of antidepressants and structured questionnaires to measure direct and indirect costs throughout the study. To evaluate the algorithm, follow-up interviews with a subgroup of patients and clinicians will also be performed.

Based on available data,^24^ an absolute difference of 10% in all-cause dropout (primary outcome) is considered clinically relevant at 8 weeks. For a rate of 25% in the control group, compared with 15% in the experimental arm, the trial will have 80% power with 248 patients in each arm (alpha 0.05), assuming that 5% of participants will be lost to follow-up. Analyses will be conducted using Mixed Models Repeated Measures for missing data. Mixed effects logistic model will be used to explore treatment effects on dichotomous outcomes. For continuous outcomes, mixed effects linear model will be used to estimate treatment effects with a baseline severity included as a covariate.

The Oxfordshire NHS Research Ethics Committee will review the trial protocol. Before the screening visit, participants will receive an information sheet with all information about the study and will provide informed consent. The Trial Management Group and the Trial Steering Committee will independently monitor the conduct of the trial.

## Patient and public involvement and participation

Patients and members of the public will be actively involved throughout the research project, as follows:

- *Project management:* management of the research project, being part of the steering committee and the advisory group.
- *Study design*: contributing to design and layout of the treatment algorithm, and the trial protocol.
- *Design of informed consent materials:* developing understandable consent and information sheets for people taking part in the trial.

*Member of the research team:

- Participation in carrying out some aspects of the research (eg, focus groups).
- Participation in interpretation of results of the research.

*Dissemination of findings: (eg, contributing to the writing-up and presentation of the outcomes from the project) and making sure the research is reported is communicated clearly and appropriately.

All patient and public involvement and engagement (PPIE) activities will be organised in collaboration with the Patients and Research Group, a group comprised of patients, carers and public members together with staff members from across mental health research and care within the NHS in Oxfordshire and Buckinghamshire, under the Oxford Health Biomedical Research Centre PPIE programme.

## Discussion

This is a clinically relevant project, coordinated by an international team of experts and with important implications for patients in the NHS. The main potential barriers to success are the access to individual-level data (for the algorithm) and poor recruitment (for the trial). Access to pharmaceutical company data proved challenging in the previous projects, but recent changes in policy have helped.^31^ Obtaining IPD is challenging, but our work in the field and our extensive network of collaborators will materially help.^32^

Another challenge is that the data from randomised trials will be gathered via highly standardised forms, whereas observational data will be lacking in standardisation. However, some critical outcomes like drug discontinuation are the same and other measures can be shown to correspond across the apparent methodological divide.^33^ In two recent examples, observational data corroborated findings from randomised trials, increasing the precision of treatment estimates.^22^ Observational studies complement randomised evidence and address some of its limitations, such as short follow-up time, small sample size, highly selected study populations and scant information about side effects. Truly representative samples are required to identify which prognostic factors (analysed with effect modifiers from randomised trials) have real-world predictive value. Applying the methodology to individual patient data will be the key innovative step within this study.

The pilot trial cannot be run in a blind fashion. The treatment algorithm will generate a list of drugs based on the best available data from randomised trials and observational datasets, but patients and clinicians will then be able to enter their own preferences in the web-based algorithm (for instance, the adverse events they want to avoid or the specific antidepressant they prefer). The weighting of these preferences will change and personalise the final ranking of treatment options. Participants will be seen in their usual clinical setting (primary or secondary care, respectively), so that the intervention occurs in a real-world setting. The advantages of individual randomisation outweigh clustering because the algorithm is in essence personal and so the solution for one person does not generalise to another individual with different characteristics or preferences.

There are two main ethical issues arising from this project: (1) confidentiality of data and (2) the use of public data to generate intellectual property. To protect patients’ confidentiality, all data used in this project will be anonymised or pseudo-anonymised. Data from randomised trials and observational data will be subject to an agreed and binding confidentiality agreement with the study sponsor.

The project will only focus on drug treatment, rather than including psychological interventions for two reasons: (1) antidepressants are the most commonly used treatment for major depressive disorder worldwide and (2) data collected from antidepressant trials are similar and reliable enough to allow the innovative analyses that are necessary to stratify and rank treatments for each individual. However, our study will form a test-case. If shown to work adequately, the algorithm can be modified and adapted to cover different treatments (ie, psychological interventions, either face-to-face or internet-delivered), other populations of patients (for instance, children and adolescents) and other psychiatric disorders (ie, schizophrenia or bipolar disorder). It can also be used in other non-communicable diseases, where the relationship between clinicians and patients spans over a long period of time and patients’ preferences are crucial to determine which is the treatment of choice and increase adherence to treatment.
